# Effects of Government Spending on Research Workforce Development: Evidence from Biomedical Postdoctoral Researchers

**DOI:** 10.1371/journal.pone.0124928

**Published:** 2015-05-01

**Authors:** Hyungjo Hur, Navid Ghaffarzadegan, Joshua Hawley

**Affiliations:** 1 John Glenn School of Public Affairs, The Ohio State University, Columbus, Ohio, United States of America; 2 Department of Industrial and Systems Engineering, Virginia Tech, Blacksburg, Virginia, United States of America; VU University Amsterdam, NETHERLANDS

## Abstract

We examine effects of government spending on postdoctoral researchers’ (postdocs) productivity in biomedical sciences, the largest population of postdocs in the US. We analyze changes in the productivity of postdocs before and after the US government’s 1997 decision to increase NIH funding. In the first round of analysis, we find that more government spending has resulted in longer postdoc careers. We see no significant changes in researchers’ productivity in terms of publication and conference presentations. However, when the population is segmented by citizenship, we find that the effects are heterogeneous; US citizens stay longer in postdoc positions with no change in publications and, in contrast, international permanent residents (green card holders) produce more conference papers and publications without significant changes in postdoc duration. Possible explanations and policy implications of the analysis are discussed.

## Introduction

In 2013, the US government spent more than 130 billion dollars to fund research activities in basic and applied areas in fields as diverse as biomedical sciences, energy, space, environment, and defense [1]. The funding was distributed to research institutions and individuals through several government organizations, such as the National Institutes of Health (NIH) and the National Science Foundation (NSF) [2]. Government research funds are intended to foster research discoveries through training and recruiting researchers as well as developing and equipping related facilities and improving research productivity. Success in these policies can be evaluated by the extent to which funding allocations result in actual outcomes in the forms of science advancement, major breakthroughs, and workforce development, which all ultimately benefit the public.

While we intuitively expect that more government spending in the form of providing research grants should increase the population of researchers, there is a range of mixed arguments about possible effects on each individual researcher. On the positive side, more funding is expected to increase the productivity of individuals through helping equip research laboratories, and providing more opportunities for high-risk, but potentially high-outcome research [3–4]. More funding also helps career advancement in academia and provides opportunities for attending conferences, networking, and collaborations [5–6]. It can also result in more and longer professional training programs which can potentially lead to knowledgeable and productive individuals [7]. With less funding, researchers end up competing for limited resources and consequently spending a major portion of their time writing and submitting grant proposals which negatively affects their ‘actual’ research activities [3,8–9]. Besides, with limited resources, researchers are likely to feel frustrated and dissatisfied by lack of job security [10].

On the other hand, there has been a range of arguments about potential unintended consequences of increasing funding. In the long term, with more funding, universities recruit more students, who will later join the pool of funding applicants and decrease the likelihood of winning a grant for each individual [3,11]. At the institutional level, universities expand their capacities with expecting funding arrival and in turn put more pressure on their faculty and research staff to bring funding [11–12]. Moreover, more funding can potentially result in longer postdoctoral careers and fewer incentives to find permanent positions [13]. Thus, in a tight economy, more funding might only result in creating temporary “holding” positions rather than research opportunities. This can also send the wrong signals to potential PhD applicants. Previous research has shown that interest in PhD programs depends on the market conditions at the time of students' enrollment rather than the expected conditions at the time of graduation [14]. When prospective students see that most graduates are hired, even though some are in temporary positions, they might not accurately understand job market conditions, or might expect funding-based positions to be available for them at the time of graduation.

Given the huge investments that governments make in supporting research activities, the fuzzy and mixed evidence of effects of funding on research outcomes and science workforce development raises important questions for science policy scholars. Understanding changes in research productivity and outcomes is complex. One major reason is that researchers are not homogenous individuals; they differ in their capabilities, incentives, institutions, work colleagues, and family and personal conditions. The latter factors can in fact make a huge difference, given that preferences and limitations that individuals with different family and immigration statuses face are different. As shown in previous studies, such personal factors affect individual decision making [15–16]. Furthermore, established versus young researchers can behave differently in response to changes in funding.

Taking individual factors into account, we investigate effects of change in research funding on researchers’ productivity. In order to provide a more focused analysis and decrease several sources of variation in researchers’ behavior, activities, and incentives, we narrow down our analysis to postdoctoral researchers (postdocs)—a specific subpopulation of researchers who are a relatively young and unestablished body of the workforce. Performance of postdocs is arguably central for future performance of science and engineering, and due to the growing number of this population, there has been an increasing concern about productivity and career prospects of this population [17]. We will consider effects of several individual level factors that influence postdocs’ performance including their citizenship status.

In this study, we take the doubling of NIH funding in 1998–2003 as a unique natural experiment opportunity to analyze change in productivity of postdocs. The event is described in the methodology section in more details. We will later discuss policy implications of our findings.

## Postdocs and the Question of Productivity

There are different stages of research workforce development, one of which is postdoctoral training. A postdoc is an individual that holds a doctoral degree and works in a *temporary* research position, usually in a university, research center, or other research institutions and laboratories [18]. As stated by the National Postdoctoral Association, postdoc positions are aimed at helping individuals acquire technical and professional skills that they need in order to pursue research careers [18].

The majority of postdocs are interested in tenure-track academic jobs [19]. The job market for tenure-track positions has been very competitive since the rate of job openings is less than the rate of PhD graduation [13,20]. As a result, every year some of the individuals who do not land academic positions take temporary postdoc positions to do more research and improve their chances in the future job market. This trend has resulted in a continuous growth in the number of postdocs, which has almost doubled since 1995 with domestic researchers staying longer in postdoc positions [7]. These days, most individuals stay three or more years in postdoc positions [21] and at the end of the experience, only 21% of them land tenure-track positions [19].

Here, we explore possible effects of change in research funding on postdocs.

### Holding and Training Positions

In order to investigate potential effects of an increase in research funding on postdocs’ productivity, we should consider career characteristics of postdoc positions. The literature considers postdoc positions as both *holding positions* and *training opportunities*.

Postdoctoral positions play the role of *holding positions* and exist because there is a shortage of tenure-track opportunities [13]. They help individuals take advantage of relatively low-paid academic positions while they seek better permanent positions [22–23]. When due to economic conditions, permanent jobs are limited, researchers more often take such opportunities [24–25]. Several recent studies argue that permanent faculty positions have become very difficult to find. Every year many researchers extend the duration of postdoctoral positions, and as a result the number of postdocs has been increasing [13,23,26]. Of course, waiting durations depend on the availability of funding; in the absence of funding and with fewer postdoc positions, people have to make a choice quickly and probably leave academia.

If we follow this line of reasoning, one expects that more government spending should expand the holding positions, resulting in longer postdoctoral careers. With more funding, individuals will have more opportunities to wait in the line before their desired jobs come up. Principal investigators are also likely to keep their trained and experienced postdocs when financial resources are more available. Thus we propose to investigate the following hypothesis:

*H1*: *More funding results in longer postdoc durations*.

On the other hand, postdoc positions are *training opportunities*. There are various studies that provide evidence on the positive effects of postdoctoral training [27], which suggests more investments and government spending in this regard. Postdoc opportunities can help individuals get additional training and get prepared for new research activities [28]. Studies show that postdoctoral training improves long term productivity of researchers reflected in their higher number of publications [29] and shorter time to secure first research grants [30]. Su (2013) shows that long postdoctoral research positions up to three years increase the chance of landing prestigious academic careers [31]. Success in the job market, publications, and grant applications is critical in researchers’ promotion in academic positions and getting tenured [4–6]. Thus, postdoc trainings can help long-term achievements of individuals in academia.

Postdocs can also help team learning. They usually work in research teams with students, professors, and other staff members. In comparison to other team members, especially students, postdocs are already trained at a PhD level and can quickly start contributing to research projects. They can help by supervising students and managing different parts of research projects [13]. Furthermore, through postdoc opportunities, young scholars usually learn to direct groups of research students and help collaborative activities [17]. Their performance, thus, influences research teams and contributes to higher team performance [32].

Therefore, we expect that more funding should result in increased positive effects of postdoctoral positions. With more and larger grants, postdocs feel more job security and can take higher risk initiatives. Also, with more funding resources, postdocs can attend more conferences and training sessions (such as method workshops), work in better equipped laboratories, and work in larger inter-disciplinary teams. Especially in experimental fields, research activities are costly and resource dependent. Thus, we offer the following hypothesis:

*H2a*: *More funding increases average productivity of postdocs*.

While we intuitively expect to find support for the positive effects on productivity (H2a), there is a possibility of observing a reverse relationship between funding and average productivity due to a set of secondary effects. For example, with more funding it is likely that more new postdocs will be hired. More postdoc openings can potentially mean weaker competition which can lead to lower quality postdocs who might not be as productive as previous ones. Furthermore, in short term, new hires can take more time from their supervisors and other team members to catch up and get initial training if needed. We therefore offer a competing hypothesis:

*H2b*: *More funding decreases average productivity of postdocs*.

### Asymmetric Effects of Funding

The complexity of understanding how researchers in general and postdocs in particular react to change in funding goes beyond difficulties of predicting how the population changes and whether in aggregate it becomes weaker or stronger. In fact, we should make sure that one does not assume that the population of postdocs is a homogenous population with similar preferences. These individuals differ in their capabilities, incentives, institutions, and family and personal conditions. Some of these factors can influence how researchers’ productivity changes when more resources are provided. While in our analysis we will control for various individual factors, such as family status, age, and institutions, we hypothesize that the effects of change in funding can be asymmetric when it comes to citizenship status.

US citizens may react differently from permanent residents (green card holders) when more resources are provided, and they all might react differently in comparison to temporary residents (visa holders). Former studies have shown that students or researchers with different citizenship or residency statuses behave differently [7,15,33]. Heterogeneities in preferences of these groups are rooted in their varying *opportunities and environmental restrictions*, *backgrounds*, and the *selection processes* they have faced.

In terms of opportunities and environmental restrictions, temporary residents are the most restricted individuals. Temporary residents face visa-related limitations which affect duration of stay in the US as well as the types of research positions that they can take. For example, there is a six-year cap for H1B visas which influences how long visa holders can remain in a postdoc position. As a result, temporary residents might have more incentives to move on to permanent jobs which are more likely to facilitate their transition to a permanent residency status. In simple words, due to visa restrictions, postdoc positions are by nature temporary positions for visa holders, but they can turn to quasi-permanent positions for other groups who face little or no residency limitations. Thus, we specifically expect that more funding should increase postdoc duration for US citizens and permanent residents as stated in the following hypothesis:

*H3*: *For US citizens and international permanent residents*, *more funding results in longer postdoc durations*.

In terms of educational background, US citizens are different from the other two groups since they are highly likely to have gotten their high school and undergraduate studies in the US. International citizens usually complete their high school and undergraduate studies in their home countries. Thus, one might expect citizenship to be a proxy for different educational backgrounds. Whether or not the difference in high school and undergraduate backgrounds of internationals versus US citizens makes one group more competitive than the other is an empirical question.

In terms of selection processes, permanent residents and temporary residents have been through several self-selection stages. The decision to take the risks of short term or long term immigration has potential dollar and social costs for individuals. In particular, permanent residents experience a few more selection processes; for example, there is an implicit analysis done to quantify the potential green card holders’ productivity. However, when we look at US citizen researchers, we are talking about the whole spectrum.

Environmental restrictions can also incentivize productivity for temporary residents. NSF reports that more than 77% of international PhD students in the US are interested in staying in the US after graduation [34]. This group should have more incentives to use every opportunity for improving their performance in order to find permanent positions.

Thus, due to the differing backgrounds and selection processes for international residents (temporary and permanent) and US citizens, as well as restrictions for temporary residents, we expect international workers to have more incentives to improve their performance than citizens, and to benefit the most from available resources. This can be reflected in their productivity measures. Thus, we offer the following hypothesis:

*H4*: *For international permanent residents and temporary residents*, *more funding results in higher productivity*.

## Methodology

### The Doubling Event

In 1997, the Congress doubled NIH budget in a very short period of five years, from $13.6 billion in 1998 to $27.1 billion in 2003, responding to higher demands for scientific studies in health sector [35]. The goal was to significantly boost research activities in biomedical fields and to increase the domestic capacities for scientific research, including maintaining and improving research infrastructure (such as laboratories) and empowering research workforce development.

The doubling event was a surprise [36–37]. Historically, predicting annual research budget in the US has been very difficult [11–12]. One reason is that every year all allocated budget should be spent and cannot be carried over to the next year [8,12]. Furthermore, there are always last minute decisions that the government and the congress should make in order to balance the whole budget, which can affect research spending, a recent example being the research budget sequestration [10]. Specifically, in regards to the doubling event, the change in budget was huge and unique in the history of US science which made it further unpredictable [36]. Evidence suggests that the biomedical science community in general and biomedical postdocs in particular did not predict the huge size and the short term period of change in the budget [36–37].

The event had several immediate impacts, as depicted in Table 1. In comparison to other fields, the ratio of NIH funding to all other federal research funding in the US increased dramatically from 0.54 to 0.81 in 2001 and 0.92 in 2003. In this period, the average grant size increased by 23% (inflation-adjusted), giving more spending flexibility to principal investigators. The number of awards increased from 7,080 to 10,393 in 2003. In the same time period, the number of applications for research project grants (RPG) in NIH also increased from an average of 24,355 to 34,710 in 2003, maintaining the overall success rate of applications (the ratio of grant applications funded) around 30% in this time period. Change in funding also resulted in more PhD admission in biomedical sciences; in five years the number of admissions increased by 23% [38].

**Table 1:** Major funding measures within NIH in 1995–2003.

**Time period**	**Ratio of NIH budget to other federal research spending**	**NIH Budget (Billions, 1995 $)**	**Grant Size (Thousands; 1995 $)**	**Awards**	**Grant Applications**	**Success Rate**
**1995–1998**	0.54	12	255	7,080	24,355	29%
**2003**	0.92	22	313	10,393	34,710	30%
**Percentage growth**	**70%**	**83%**	**23%**	**47%**	**43%**	**3%**

The doubling event, both in terms of numbers and the created attention, has been a major shift in US biomedical research. At the outcome level, it is expected that such a huge surge in research funding in a relatively short time period should have major impacts on the biomedical research community. The event provides a unique natural experiment context to study effects of rapid change in funding on research enterprises and workforce. We focus on the specific question of productivity as affected by funding, and study the effects of doubling on postdocs in biomedical fields. The dataset and variables are described in the following.

### Data

Our analysis is based on data from the 1995, 2001, and 2003 of Survey of Doctoral Recipients (SDR), conducted by NSF. The dataset is a nationally representative sample of the population of PhD graduates living in the US. Participants’ consent is obtained through NSF. The dataset is anonymized and de-identified by NSF, and is made publicly available for research purposes upon request from NSF. The dataset is IRB exempt by behavioral and social sciences committee of IRB at the Ohio State University.

For the purpose of our analysis, we draw data on current postdoctoral researchers from these datasets. We code the following fields as related to biomedical sciences: biochemistry and biophysics, biology, cell and molecular biology, genetics, animal and plant, microbiological sciences and immunology, nutritional sciences, pharmacology, human and animal, physiology and pathology, human and animal, and zoology [38].

### Outcome Variables

We use four outcome measures. The first measure is *time in the latest postdoc*. This variable is calculated by using the starting month and year of the recent postdoctoral job and the time that the survey is conducted. The second measure is *time since graduation*. Time since graduation is calculated by using the awarding month and year of US PhD and the time that the survey is conducted. The third measure is the number of papers presented at conferences during the last five years. The fourth measure is the number of (co)authored articles accepted for publication during the last five years. Table 2 reports descriptive statistics for the outcome variables.

**Table 2:** Descriptive Statistics of Four Outcome Measures: 1995, 2001, and 2003.

**Variable**	**Sample size**	**Mean**	**Standard deviation**	**Min**	**Max**
**Time in Latest Postdoc (Months)**	3,669	27.54	32.72	0	483
**Time Since Graduation (Months)**	3,669	44.25	46.67	10	544
**Number of Conference Papers**	3,669	6.45	6.88	0	96
**Number of Publications**	3,669	5.41	5.80	0	96

Note: For descriptive statistics of all variables, see S1 Table. October was the survey reference month in 2003, and April was the survey reference month in 1995 and 2001.

Source: NSF SESTAT Data, 1995, 2001, and 2003 Survey of Doctorate Recipients (SDR) (sestet.nsf.gov).

### Individual Controls

Apart from the four variables mentioned, we control for age, gender, race, marriage, children, working hours, cohort, and research focus. These variables are provided in the survey. The variable *research focus* is a dummy variable that is set to be *one* for any respondent that works in basic or applied research, and *zero* otherwise.

### Organizational Controls

We use the 1994 Carnegie classification code to define institutional rank scale. Postsecondary education institutions are classified as following: Research University I, Research University II, Doctorate Granting I, Doctorate Granting II, and others (Master’s Universities and Art College I, II, Baccalaureate College I, II, Associate of Arts College, Professional Schools and Specialized Institutions). We include this measure because of the assertion that higher ranking university postdoctoral researchers have more chances to contact and work with other professors in their university or other universities. Moreover, as NIH grants are highly concentrated geographically and institutionally, it is important to control for institutions in the analyses to allow for comparison by organization.

### Model

We conduct two rounds of analyses. In the first round, we look at the whole sample of postdoctoral researchers regardless of their citizenship. In the second round of analysis, we run models separately for different citizenship statuses (US citizens, permanent residents (green card holders), and temporary residents (visa holders)).

Since doubling NIH funding started in 1998, we compare data in 1995 with 2001 and 2003. Due to NIH’s mission, its funding is more concentrated among biomedical researchers than other fields. More funding is expected to increase resources that ultimately improve productivity of scientists in biomedical sciences than scientists in other fields. We will compare the productivity of postdoctoral researchers in biomedical fields against postdoctoral researchers in non-biomedical fields in a classic difference-in-difference analysis.

Specifically, we run the following model with four dependent variables.

$$y_{it}=\beta_{0}+\beta_{1}BiomedicalField_{it}+\beta_{2}DoublingFunding_{it}+\beta_{3}BiomedicalField_{it}\times DoublingFunding_{it}+k_{it}+\varepsilon_{it}$$ (1)

In this equation, *y*_*it*_ is the outcome of interest for individual *i* given time *t*. The dummy variable *BiomedicalField* captures possible differences between the treatment (biomedical postdocs) and comparison groups (non-biomedical postdocs). The variable *DoublingFunding* is equal to 1 for the second time period (2001 and 2003), and equal to zero for year 1995. The variables *k* represent other control variables (age, gender, race, marriage, children, research activity, working hours, research focus, cohort, and institutional rank). Finally, duration of postdoctoral career is added as a control variable when we study conference papers and publications as dependent variables. In a difference-in-difference analysis, the coefficient of the interaction term (***β***_***3***_) represents the effect.

We use the ordinary least-square regression for the first two dependent variables and test the first and third hypotheses. For testing the second and forth hypotheses, since the dependent variables (number of conference papers and publications) are non-negative integers with a skewed distribution (there are many 0 or 1 values), we conduct negative binomial regression analysis.

## Results

### Effects on the Whole Population

**Fig 1:**
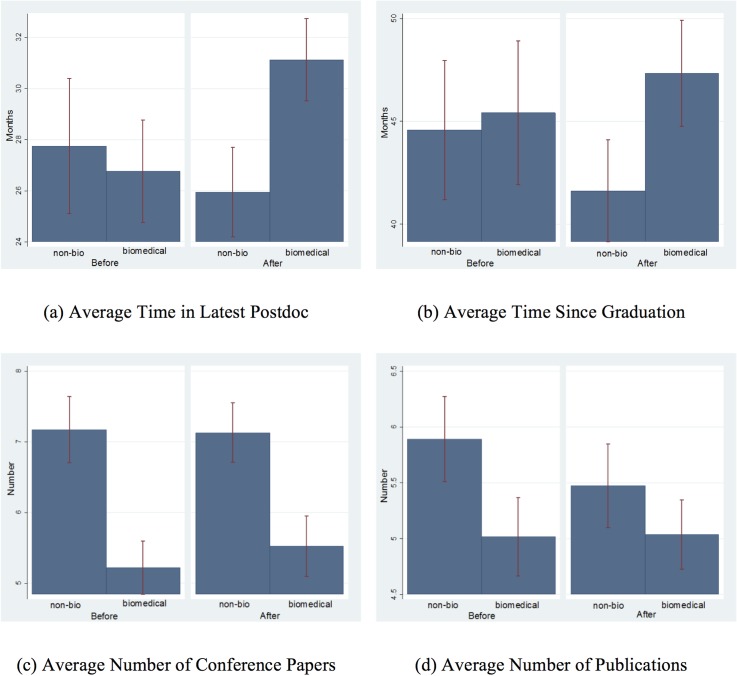
Change in major variables in biomedical and non-biomedical fields after doubling NIH funding. Error bars represent 95% distribution.

Graphs in Fig 1 compare the dependent variables before and after doubling for non-biomedical and biomedical postdocs. In Fig 1A, the *average time in latest postdoc* in biomedical fields increases about four months after doubling funding. In non-biomedical fields, this number decreases by about two months. As Fig 1B shows, the variable *time since graduation* is increased in biomedical fields by about two months after doubling funding, while in non-biomedical fields, it decreased by about three months. Fig 1C shows that the average number of conference papers behaved similarly in biomedical and non-biomedical fields after doubling. Fig 1D shows a relatively steady number of publications per person in biomedical fields and a declining trend in non-biomedical fields.

Table 3 depicts effects of doubling NIH funding on the whole population of postdoctoral researchers. It summarizes the results of regressions reporting ***β***_***1***_, ***β***_***2***_ and ***β***_***3***_ from Eq 1. ***β***_***1***_ (the coefficient of *Biomedical Field*) represents the overall difference between treatment group and control group, ***β***_***2***_ (the coefficient of *Doubling Funding*) represents the overall trend for all fields, and ***β***_***3***_ (the coefficient of Doubling Funding*Biomedical Field) represents the main effect from a difference-in-difference analysis: the effect of doubling funding for the treatment group controlling for secular and between field effects.

**Table 3:** Difference-in-Difference estimates for the Doubling Funding Effect.

Change of pre and post of Doubling Funding	DV: Time in Latest Postdoc	DV: Time Since Graduation	DV: Conference Papers	DV: Published Articles
*ALL*				
Biomedical Field (***β***_***1***_)	-0.85	0.86	-0.35***	-0.24***
	(1.47)	(1.28)	(0.04)	(0.04)
Doubling Funding (***β***_***2***_)	9.95***	71.79***	-0.17**	0.05
	(2.87)	(2.50)	(0.09)	(0.08)
Difference in Difference (***β***_***3***_)	5.76***	3.93**	0.03	0.09
	(2.02)	(1.76)	(0.06)	(0.06)
Observations	3,664	3,664	3,664	3,664
Adj R-squared / Log likelihood	0.20	0.71	-10499.99	-9787.06

*** p<0.01,

** p<0.05,

* p<0.1

Note: Standard errors are presented in parentheses below coefficient estimates. DV stands for dependent variable. Control variables include age, gender, race, marriage, children, working hours, research focus, cohorts, time in the last postdoc (only when DV is conference papers or published articles), and institutional rank of the organization where researchers got their first US S&E or health PhD. Data source: NSF SESTAT Data, 1995, 2001, and 2003 Survey of Doctorate Recipients (sestet.nsf.gov).

The results show that ***β***_***3***_ is significant in two of the analyses. Comparing with non-biomedical researchers, *time in the latest postdoc* has significantly changed after the start of the funding policy. Postdoctoral researchers in biomedical fields stayed around six more months (p <.01) in comparison to non-biomedical researchers. Moreover, after doubling funding policy, *time since graduation* of doctoral recipients in biomedical fields was increased by approximately four months compared to non-biomedical fields (p<.05). As shown in Table 3, the numbers of conference papers and publications per person have not significantly changed after the doubling policy.

Among the control variables (not shown in Table 3), gender is significant in the third and fourth models (p<0.1), implying males write more papers and go to more conferences. Postdocs who are parents are also less likely to stay as long in postdoc positions, which can be due to lower average wage of such positions and the need to take permanent jobs.

In summary, the analysis shows that doubling funding increased time in the recent postdoc position and time since graduation among biomedical postdocs (support for hypothesis H1) with no observable effects on productivity (no support for hypotheses H2a or H2b). To examine the robustness of our findings, we conduct two additional analyses with the same data reported in the supporting information (S2 and S3 Tables). First, we limit the data points to individuals who have government funding and, second, use different weights provided in the dataset. Similar effects appear; variables related to postdoc duration increase in treatment groups with no significant change in conference papers and publications.

### Effects on Different Citizen Groups

Table 4 summarizes the main results of the analysis on different citizen groups of postdocs. We control for a wide range of individual and organizational variables as described in the method section.

**Table 4:** Difference-in-Difference estimates for the Doubling Funding Effect.

Change of pre and post of Doubling Funding	DV: Time in Latest Postdoc	DV: Time Since Graduation	DV: Conference Papers	DV: Published Articles
***US***				
Biomedical Field (***β***_***1***_)	-2.73	0.70	-0.34***	-0.21***
	(1.90)	(1.65)	(0.05)	(0.05)
Doubling Funding (***β***_***2***_)	5.73	66.23***	-0.01	0.06
	(3.55)	(3.08)	(0.10)	(0.09)
Difference in Difference (***β***_***3***_)	7.99***	4.93**	-0.03	0.02
	(2.62)	(2.27)	(0.07)	(0.07)
Observations	2,693	2,693	2,693	2,693
Adj R-squared / Log likelihood	0.20	0.70	-7626.58	-7111.67
***GREEN CARD HOLDER***				
Biomedical Field (***β***_***1***_)	1.66	-0.87	-0.43***	-0.30***
	(2.07)	(1.25)	(0.10)	(009)
Doubling Funding (***β***_***2***_)	22.92***	78.75***	-0.82***	-0.19
	(4.56)	(2.76)	(0.24)	(0.21)
Difference in Difference (***β***_***3***_)	3.26	3.49*	0.37**	0.36**
	(3.25)	(1.97)	(0.15)	(0.15)
Observations	492	492	492	492
Adj R-squared / Log likelihood	0.21	0.85	-1411.59	-1332.04
***VISA CARD HOLDER***				
Biomedical Field (***β***_***1***_)	7.75***	0.62	-0.15	-0.27*
	(2.43)	(1.54)	(0.15)	(0.15)
Doubling Funding (***β***_***2***_)	-7.90*	92.38***	0.14	0.53
	(8.17)	(5.18)	(0.5)	(0.48)
Difference in Difference (***β***_***3***_)	-5.73*	0.45	-0.13	0.22
	(2.97)	(1.88)	(0.18)	(0.18)
Observations	479	479	479	479
Adj R-squared / Log likelihood	0.18	0.77	-1395.33	-1286.60

*** p<0.01,

** p<0.05,

* p<0.1

Note: Standard errors are presented in parentheses below coefficient estimates. DV stands for dependent variable. Control variables include age, gender, race, marriage, children, working hours, research focus, cohorts, time in the last postdoc (only when DV is conference papers or published articles), and institutional rank of the organization where researchers got their first US S&E or health PhD. Data source: NSF SESTAT Data, 1995, 2001, and 2003 Survey of Doctorate Recipients (sestet.nsf.gov).

The first column in Table 4 shows the effects of increased funding on the time in the latest postdoc. As depicted, the duration has increased for US citizens as a result of the doubling policy by about eight months (p <.01). Change in *time in recent postdoc* among permanent residents is not statistically significant and for visa card holders is in a negative direction and modestly significant (p <.10). The second column in Table 4 shows the effects of more funding on *time since graduation* of postdocs. The variable has increased for US citizens, approximately by five months (p <.05). Overall, the analysis shows that as a result of doubling funding, US citizens wait longer in the pipeline in postdoc positions.

The third column in Table 4 reports results for the average number of conference papers. Permanent residents are the only ones that show a significant change in number of conference papers per individual (p <.05). The fourth column in Table 4 shows change in publications. Again, permanent residents show a significant positive change (p <.05). We also estimated the size of the effect by looking at incidence-rate ratios. The analysis reveals that the magnitude of the effect is meaningful: for permanent residents, the numbers of conference papers and publications per person have increased by 44.4% and 43.7% as results of doubling. The analysis on US citizens does not show any significant change in conference papers and publications despite them staying longer in their latest postdoc positions.

In summary, in regards to the effects of doubling policies on postdoctoral researchers, our analysis shows that:

1. More funding resulted in US citizens staying longer in recent postdoctoral positions and extended time since graduation, with no significant effect on other groups (support for hypothesis H3 for US citizens only).
2. More funding increased number of conference papers for permanent residents with no significant effect on other groups (support for hypothesis H4 for permanent residents only).
3. More funding increased number of publications for permanent residents with no significant effect on other groups (support for hypothesis H4 for permanent residents only).

Similar effects can be found in the sensitivity analysis reported in the supporting information (S2 and S3 Tables), which shows an increase in the number of both conference papers and publications for permanent residents and longer postdocs for US citizens.

## Discussion and Conclusion

We analyzed the effects of government research spending on researchers’ productivity by focusing on the population of postdoctoral researchers in biomedical sciences and using data from Survey of Doctorate Recipients. We specifically focused on analyzing changes in productivity of postdocs before and after the US government’s decision to increase NIH funding in 1997. The main hypothesis was that, with more funding, productivity of researchers should increase as they are able to attend more conferences, are better equipped with research facilities and laboratories, and have more supporting students and staff in their teams. Productivity was measured in terms of number of publications and conference papers per researcher. In addition, we looked at the duration of postdoctoral training by focusing on time in latest postdoc positions and time since graduation. The analysis was directed toward understanding the effects on the whole group of postdoctoral researchers in biomedical sciences and on different citizen groups, hypothesizing that the effects might be different for domestic versus international researchers.

Our results show little change in productivity but more significant changes in duration of postdocs and waiting time in the pipeline until landing permanent positions. In comparison to non-biomedical fields, more government spending has resulted in longer postdoc durations. It seems that the first reaction to increase in funding for postdocs is to stay in their current positions, and for supervisors is to keep their currently trained and experienced researchers. One reason for staying longer in a postdoc position despite the relatively lower pay is that individuals can wait for and seek their desired permanent position (which might be a tenure-track academic position) for a longer time period. This observation corroborates with theories that consider postdoc positions as holding positions.

When the population of analysis is segmented by citizenship, we find heterogeneous effects on different groups of biomedical researchers based on their citizenship. Specifically, US citizen postdocs stayed longer in their recent postdoc positions while their productivity was unchanged. In contrast, international permanent residents improved their productivity without significantly extending their postdoc duration. Overall, the results support the idea that different groups of researchers might react differently to change in level of funding due to their differing preferences.

This study contributes to the studies of research workforce development, and our findings corroborates with recent argument about systematic problems in science education especially in biomedical fields [7–13,39]. Our study is different from others since it moves the focus from performance of the population of scientists to individual level productivity. We also show how the reactions of the research community can be complex and asymmetric to change in funding. In other words, our results imply that the question of “what are the effects of change in funding” should be supplemented with “for which sub-population of researchers.”

This study has several limitations that suggest new avenues of research. While one of the richest available data sources is used in this study, we cannot rule out all potential variables affecting postdoctoral researchers’ behavior. We have tried to use most of the available variables at the individual and institutional level to control for a wide range of effects. In terms of productivity, we narrowed down the concept to the more objective variables of number of publications and conference papers. We acknowledge limitations of this operationalization. One would benefit from having data on quality of papers, journals’ rankings, and impacts (in terms of citations or other measures) in their analysis. These limitations suggest cross-checking the results with other data sources and other methodological approaches to better inform policy makers [40–41]. Furthermore, our analysis was focused on the time frame of 1995 to 2003. One would benefit from analyzing longer-term effects, maybe over a decade. This will require disentangling effects of funding on PhD admission decisions and on PhD training from sole effects on postdocs’ productivity, which can be explored in future research.

### Policy Implications

As noted in the introduction, identifying “policy tools” to increase the productivity of postdoctoral researchers is a critical question for US science and workforce development policy. The primary tool for the federal government to improve research output is to supply more resources through federal agencies such as NIH and NSF. However, aggregate levels of funding seem to be relatively blunt policy tools, in that they may increase resources but do not have much impact on the distribution of funds or productivity of researchers. Some of the key questions that policymakers should consider are: Who gets the funding? Are some researchers benefiting more than other groups? What fields are being funded?

Another implication is the need to consider multiple measures of performance for research policy evaluation. This analysis shows that dramatic increases in funding led to changes in the amount of time postdoctoral researchers serve in these roles, providing validation of a sort for policy makers that more funding can lead to increased opportunity to conduct scientific research. However, on the outputs side, simply increasing funding does not appear to have accelerated the publication or presentation of papers by each biomedical scientist. This is despite increased opportunity to do research. The reality is that the quality of research is a much more difficult outcome to manipulate through policy. Quality science is generally conceptualized as both the result of consistent effort over time, as well as flashes of brilliance and discovery [42]. In sum, we should judge the effectiveness of these policies based on the science output as well as quality of the results.

Moreover, it seems funding policies should be supplemented by other policy actions in order to lead to the desired goals. In other words, increasing funding is not a “silver bullet.” If more funding mainly results in longer postdoc positions, one might doubt if such a reaction from the science community is optimal while many new PhD graduates are looking for postdoctoral training positions. At the end, the number of tenure-track positions is limited and has been constant in the past years, so longer postdocs might translate to longer waiting times before eventually deciding to leave academia for industry—an inefficient policy.

Furthermore, our study provides evidence that the reaction of domestic and international researchers to change in research funding varies. US science policy has depended for decades on international researchers [33]. A substantial fraction of researchers in virtually every science sub-discipline were educated overseas and moved to the US either for doctoral or postdoctoral training [43]. Visa policies restrict non-native researchers’ job mobility, leading to potentially different motivations and providing support for the hypothesis that international researchers behave differently and might respond in unique ways to increases in government funding [15,43]. This implies that the science community is not homogenous in preferences and the same policy can have different results for different groups of the population, a fact that policy makers should consider.

Moving forward, the US government is facing increasing resource pressures and, after the American Recovery and Investment Act in 2009, is trying to readjust the science community to lower levels of resources for basic and applied work. It is necessary to come up with better policies that make science funding more effective in output and quality metrics.

## Supporting Information

S1 Table Description of variables. This table complements Table 2 in the paper by presenting descriptive statistics, definitions, and sources of variables.
(PDF)

S2 Table Difference-in-Difference estimates within only government-funded postdocs. We report extra analysis to demonstrate robustness of our main results (Tables 3 and 4) to a major change in assumptions. S2 Table limits the sample size to only individuals who have government funding (excluding postdocs with no government funding).
(PDF)

S3 Table Difference-in-Difference estimates, weighted. We report an analysis to demonstrate robustness of our main results (Tables 3 and 4) to another major change in assumptions. This table uses a different survey weight system (provided in the dataset).
(PDF)
