# Supplementary material for: Effects of Government Spending on Research Workforce Development: Evidence from Biomedical Postdoctoral Researchers
Source: PLoS One. 2015 May 1;10(5):e0124928. doi: 10.1371/journal.pone.0124928 (PMC4416806; doi:10.1371/journal.pone.0124928)
Supplement: S1 Table — This table complements Table 2 in the paper by presenting descriptive statistics, definitions, and sources of variables. (PDF) [file pone.0124928.s001.pdf]

**Table S1. Description of variables**

| Variable | No. of observations | Mean | Std. Dev. | Min | Max | Definition and Source |
|---|---|---|---|---|---|---|
| Age | 3,669 | 35.06 | 5.92 | 24 | 72 | “Number of years has been alive” |
| Gender (Male) | 3,669 | 0.58 | 0.49 | 0 | 1 | Types of Gender (Male, and Female) |
| Race (White) | 3,669 | 0.59 | 0.49 | 0 | 1 | Types of races (White, Black, Asian, Hispanic, and Others) |
| US | 3,669 | 0.74 | 0.44 | 0 | 1 | Types and Status of Citizenship (US citizen, GREEN card holders, and VISA card holder) |
| Biomedical | 3,669 | 0.39 | 0.49 | 0 | 1 | Field of major for first US S&E or health PhD (Biomedical, and Non-Biomedical) |
| Number of Conference Papers | 3,669 | 6.45 | 6.88 | 0 | 96 | The survey question is "How many papers have you (co)authored for presentation at regional, national or international conference?” since survey month and year. The survey of 1995 was asked ‘since April 1990’, the survey of 2001 was asked ‘since April 1995’, and the survey of 2003 was asked ‘since October 1998’. |
| Number of Publications | 3,669 | 5.41 | 5.80 | 0 | 96 | The survey question is "How many of articles (co)authored by you, have been accepted for publication since survey month and year?”. The survey of 1995 was asked ‘since April 1990’, the survey of 2001 was asked ‘since April 1995’, and the survey of 2003 was asked ‘since October 1998’. |
| Time in Latest Postdoc | 3,669 | 27.54 | 32.73 | 0 | 483 | Subtract from "Month and year of Survey" to "Month and year of Start this Postdoc Job" |
| Time Since Graduation | 3,669 | 44.25 | 46.67 | 10 | 544 | Subtract from "Month and year of Survey" to "Month and year of award of first US PhD" |
| University Rank 1 | 3,669 | 0.76 | 0.42 | 0 | 1 | Carnegie classification for school awarding first US S&E or health PhD: 1994 Carnegie code (Research University I, Research University II, Doctorate Granting I, Doctorate Granting II, and Others) |
| Work Activity (Research) | 1,807 | 0.72 | 0.45 | 0 | 1 | Work Activity Spend most hours on in principal job ('Basic or Applied research study to gain sci. knowledge to meet recognized need', and 'Others such as teaching, etc.') |
| Work Hours | 3,669 | 49.23 | 10.88 | 1 | 96 | During a typical week on this job, how many hours did you usually work? |
| Graduation Year (Cohort) | 3,669 | 1995.83 | 4.00 | 1990 | 2002 | Academic Year of award for first US S&E or health PhD |
| Married | 3,669 | 0.63 | 0.48 | 0 | 1 | Marriage Status (Married, and others) |
| Children | 3,669 | 0.38 | 0.49 | 0 | 1 | "Do you have any children living with you as part of your family?" |
