# Supplementary material for: Effects of Government Spending on Research Workforce Development: Evidence from Biomedical Postdoctoral Researchers
Source: PLoS One. 2015 May 1;10(5):e0124928. doi: 10.1371/journal.pone.0124928 (PMC4416806; doi:10.1371/journal.pone.0124928)
Supplement: S2 Table — We report extra analysis to demonstrate robustness of our main results (Tables 3 and 4) to a major change in assumptions. S2 Table limits the sample size to only individuals who have government funding (excluding postdocs with no government funding). (PDF) [file pone.0124928.s002.pdf]

Table S2. Difference-in-Difference estimates within only government-funded postdocs

Change of pre and post of Doubling Funding	Time in Latest Postdoc		Time Since Graduation		Conference Papers		Published Articles	
	Effect	Standard error	Effect	Standard error	Effect	Standard error	Effect	Standard error
ALL								
Biomedical Field (β_1)	0.32	1.6	-0.08	1.39	-0.45***	0.05	-0.29***	0.05
Doubling Funding (β_2)	18.85***	3.35	70.23***	2.92	-0.17	0.11	0.11	0.11
Difference in Difference (β_3)	2.86	2.21	4.11**	1.93	0.1	0.07	0.08	0.07
Observations	2,410		2,410		2,410		2,410	
R-squared / Log likelihood	0.19		0.69		-6965.26		-6571.53	
US								
Biomedical Field (β_1)	-1.51	2.02	0.08	1.76	-0.41***	0.06	-0.23***	0.06
Doubling Funding (β_2)	15.40***	4.11	65.34***	3.58	-0.01	0.12	0.13	0.12
Difference in Difference (β_3)	5.02*	2.8	5.03**	2.44	0.02	0.08	-0.03	0.08
Observations	1,798		1,798		1,798		1,798	
R-squared / Log likelihood	0.19		0.70		-5131.57		-4855.63	
GREEN CARD HOLDER								
Biomedical Field (β_1)	3.42	2.58	-0.77	1.45	-0.66***	0.12	-0.46***	0.12
Doubling Funding (β_2)	29.87***	5.66	75.64***	3.18	-0.71**	0.29	-0.08	0.26
Difference in Difference (β_3)	-1.63	4.11	1.57	2.31	0.62***	0.19	0.49***	0.18
Observations	324		324		324		324	
R-squared / Log likelihood	0.26		0.85		-932.94		-893.79	
VISA CARD HOLDER								
Biomedical Field (β_1)	12.35***	3.51	0.48	2.05	-0.06	0.18	-0.37**	0.18
Doubling Funding (β_2)	-16.08	15.35	72.15***	8.98	-0.24	0.76	-0.82	0.8
Difference in Difference (β_3)	-10.44**	4.2	0.77	2.46	-0.23	0.22	0.37*	0.22
Observations	288		288		288		288	
R-squared / Log likelihood	0.19		0.72		-835.65		-771.92	

*** p<0.01, ** p<0.05, * p<0.1

Control variables include age, gender, race, marriage, children, working hours, research focus, cohorts, time in the last postdoc (only when DV is conference papers or published articles), and institutional rank of the organization where researchers got their first US S&E or health PhD. Source: NSF SESTAT Data, 1995, 2001, and 2003 Survey of Doctorate Recipients (SDR) (sestet.nsf.gov).
