# Supplementary material for: Effects of Government Spending on Research Workforce Development: Evidence from Biomedical Postdoctoral Researchers
Source: PLoS One. 2015 May 1;10(5):e0124928. doi: 10.1371/journal.pone.0124928 (PMC4416806; doi:10.1371/journal.pone.0124928)
Supplement: S3 Table — We report an analysis to demonstrate robustness of our main results (Tables 3 and 4) to another major change in assumptions. This table uses a different survey weight system (provided in the dataset). (PDF) [file pone.0124928.s003.pdf]

Table S3. Difference-in-Difference estimates, weighted

Change of pre and post of Doubling Funding	Time in Latest Postdoc		Time Since Graduation		Conference Papers		Published Articles	
	Effect	Standard error	Effect	Standard error	Effect	Standard error	Effect	Standard error
ALL								
Biomedical Field (β_1)	-2.79	2.13	-0.61	1.96	-0.37***	0.05	-0.25***	0.05
Doubling Funding (β_2)	2.67	7.92	74.30***	6.42	-0.21*	0.12	0.1	0.13
Difference in Difference (β_3)	7.25***	2.63	4.42*	2.25	0.09	0.07	0.1	0.07
Observations	3,664		3,664		3,664		3,664	
R-squared / Log likelihood	0.24		0.73		-169256.7		-157963.21	
US								
Biomedical Field (β_1)	-5.86**	2.83	-1.48	2.54	-0.39***	0.06	-0.22***	0.06
Doubling Funding (β_2)	-4.14	9.4	68.33***	7.68	-0.07	0.14	0.13	0.15
Difference in Difference (β_3)	10.68***	3.47	6.00**	2.93	0.05	0.09	0.03	0.09
Observations	2,693		2,693		2,693		2,693	
R-squared / Log likelihood	0.24		0.74		-117136.67		-108771.33	
GREEN CARD HOLDER								
Biomedical Field (β_1)	1.83	2.22	-1.28	1.31	-0.39***	0.11	-0.33***	0.11
Doubling Funding (β_2)	21.41**	10.65	78.33***	3.98	-0.75***	0.24	-0.18	0.27
Difference in Difference (β_3)	2.29	3.84	3.29	2.39	0.34**	0.16	0.32**	0.15
Observations	492		492		492		492	
R-squared / Log likelihood	0.25		0.86		-23879.04		-22824.09	
VISA CARD HOLDER								
Biomedical Field (β_1)	7.44***	2.8	0.31	0.87	-0.1	0.14	-0.30**	0.13
Doubling Funding (β_2)	-5.42	7.26	91.40***	8.20	-0.07	0.19	0.31	0.38
Difference in Difference (β_3)	-5.68*	3.2	0.33	1.58	-0.18	0.17	0.22	0.16
Observations	479		479		479		479	
R-squared / Log likelihood	0.23		0.76		-27052.14		-25280.27	

*** p<0.01, ** p<0.05, * p<0.1

Control variables include age, gender, race, marriage, children, working hours, research focus, cohorts, time in the last postdoc (only when DV is conference papers or published articles), and institutional rank of the organization where researchers got their first US S&E or health PhD. Source: NSF SESTAT Data, 1995, 2001, and 2003 Survey of Doctorate Recipients (SDR) (sestet.nsf.gov).
